# Transitioning from autism self-advocacy to advocating for the neurodiverse community

**DOI:** 10.4102/ajod.v14i0.1560

**Published:** 2025-04-14

**Authors:** Emile Gouws

**Affiliations:** 1 Department of Inclusive Education, College of Education, University of South Africa, Pretoria, South Africa

**Keywords:** autism self-advocacy, evocative autoethnography, co-constructors, neurodiversity, Global South

## Abstract

**Background:**

In this article, I reflect on my continuing journey of becoming an autism self-advocate and how this has extended to advocate for the broader neurodiverse community.

**Objectives:**

I aimed to provide an academic analysis of the value of autism self-advocacy with special reference to building resilience to confront discrimination and advocating for equal opportunities, support and inclusion.

**Method:**

My autoethnographic reflections are fused with those of my co-constructors to present critical incidents that defined my journey of becoming an internationally recognised advocate for autism.

**Results:**

Education has been the transformative force that has changed my life, granting me opportunities to advocate for my broader neurodiverse community on various local and international platforms and guiding me to create an ecosystem of family and professionals who continuously support me and others who are neurodiverse. My advocacy, which aims at raising autism awareness, has changed the perceptions of the community of practice as well as others with an interest in supporting and including individuals living with autism in a significant way.

**Conclusion:**

A network of care and support is required to sustain autism self-advocacy and to build on it to advocate for the broader neurodiverse community.

**Contribution:**

This demonstrates the need for educating stakeholders to raise their expectations for autistic children and adults and for teachers and psychologists to continue supporting families and children with Autism Spectrum Disorder (ASD) to realise their full human potential.

## Introduction

This article discusses my ongoing journey to being an advocate for all individuals living with autism. It presents two significant incidents that have influenced this journey, each of which was supported by my co-constructors who have played a crucial role. Autism self-advocacy is a movement that empowers individuals living with autism to change the attitudes and perceptions portrayed by society (Pellicano & Den Houting 2022). Autism self-advocacy has significantly influenced my professional and personal life. Now, on the brink of transitioning to the next chapter of my journey as a postdoctoral fellow, I aim to combine my advocacy work with academic research. My advocacy has grown to represent the broader neurodiverse community, and these support systems have helped me as an autism self-advocate towards personal growth in unique ways.

## Problem statement and study goals

Autism Spectrum Disorder (ASD) is a lifelong prevalent neuro-developmental difference that manifests through challenges in communication, social interactions and behaviour that includes rigid routines and an aversion to changes in their environment (Thwala 2018). Autism Spectrum Disorder self-advocacy is a movement in which individuals on the autism spectrum disorders advocate for themselves and fight for their rights (Ward & Meyer 1999). This attitude is based on the idea of self-determination and aims to provide an insider’s perspective on how society views and treats people with autism spectrum disorder daily (Glumbić, Đorđević & Brojčin 2022). The goal of autism self-advocacy is to bring about the necessary changes to create a more inclusive and accessible society (Leadbitter et al. 2021). Unfortunately, many individuals engage in autism self-advocacy because of the negative experiences they have faced in various societal settings, such as schools and universities. The main objective of autism self-advocacy is to challenge ableist views and stigma (Kamperman 2020). Fundamentally, many autistic people see their diagnosis as part of their identity. The focus is on the ethics of masking as a coping strategy and mental health awareness (Botha 2021).

Newell et al. (2023) reviewed 36 studies on the mental health of autistic adults. They found that 95% of the participants had suicidal thoughts, and 24% had attempted to commit suicide before receiving treatment for depression. The positive side of this study, which corresponds to my experiences, is those individuals who use self-advocacy as a vehicle to improve their self-confidence and eventually heal their depression.

In this study, I share my journey by reflecting on the events that led me to become an autism self-advocate, through which I discovered my purpose. I could not have achieved personal and academic success without the ongoing support of my ecosystem. I believe that self-advocacy is crucial for making society more inclusive and accessible. In the next stage of my journey, I am advocating for my neurodiverse community who may not have the opportunities and resources to advocate for themselves.

## Background statement

Autism self-advocacy has significantly influenced my professional and personal identity. Growing up, I believed that the purpose of my life was to contribute to society in a meaningful way. Despite the discrimination that I experienced as an autistic child because of my limited speech ability and the social and emotional challenges I encountered, I developed the courage to defy the odds and prove to the people who had judged me as a separate entity based on my differences that I could complete my PhD and become an advocate for neurodiversity rights at the highest political level (Gouws 2022). This has motivated me for the past 7 years to participate in social activities for the benefit of society by engaging with organisations that provide autistic adults with platforms to share their stories, such as nonprofit organisations, government departments and civil society in general. My focus has included advising and contributing towards different policies regarding disabilities worldwide and in Commonwealth countries to enhance accessibility for individuals from marginalised groups. Also, drawing on my experiences as a neurodiverse adult, I have striven to guide parents of neurodiverse children and young adults by providing advice, encouragement and support.

By recalling and reflecting on two critical incidents, I hope to highlight experiences that have shaped my identity as an international autism and disability rights activist. Using the lens of Bronfenbrenner’s ecological theory and objective self-awareness theory, I explain how these experiences have shaped my identity as a neurodiverse adult (Härkönen 2001). This has been a journey of personal growth, and I am motivated to continue developing and evolving my advocacy initiatives and academic skills.

## Literature review

The purpose of autism self-advocacy is to empower autistic people worldwide to take control of their lives and improve society for the better (Waltz et al. 2015). The history of the autism self-advocacy movement is a testament to the resilience and determination of the autistic community that emerged during the 1990s as a valid form of meaning and acceptance of the individual beyond diagnosis (Zuber & Webber 2019). The year 2000 brought a new development that would spark a change in the way autism was viewed. The neurodiverse movement emphasised the unique attributes and abilities of neurological differences in society (Ortega 2013) and gained momentum because of the voices, determination and advocacy of the autistic community (Petri 2018). According to the United Nations Convention on the Rights of Persons with Disabilities (UNCRPD), neurodiversity intersects with civil rights and broader disability rights. Proponents from a medical perspective view autism as a disability, and as such, the neurodiverse movement outlines the message that all individuals deserve recognition and acceptance. The common perception in the 21st century is that the neurodevelopmental profile of neurodiverse individuals is regarded as a strength and not a weakness, marking a positive shift in societal attitudes and offering hope for the future (Chapman & Veit 2020). In saying this, self-advocacy is a powerful tool that allows individuals to express their feelings and societal position. It is a means to improve facilities and current circumstances and is crucial for advancing the autism self-advocacy movement (Glumbić et al. 2022; Petri, Beadle-Brown & Bradshaw 2021).

The self-advocacy movement is influenced by cultural values and location (Ortega 2013). For instance, Waltz et al. (2015) pointed out that some cultures in Northern Europe are shaped by masculine and ableist expectations, which perceive autism traits as a form of weakness and a social problem. These perceptions influence attitudes towards autism on the continent of Africa too, especially in South Africa. Self-advocates emphasise the importance of understanding individual identity development in various settings and consider the mental health of individuals with autism, which builds resilience to persevere during difficult times (Cook & Purkis 2022).

## Theoretical approach

This academic article uses a theoretical approach based on Bronfenbrenner’s ecological systems and objective self-awareness theory. However, the chosen methodology means that the theoretical position and the author’s personal experiences are closely intertwined. My theoretical understanding and personal experiences as an autistic individual have profoundly influenced my life and continue to do so today as they shaped my purpose in life, shifting my focus from myself to advocating and empowering the neurodiverse community. Growing up, I was fortunate to have a robust support system that supported me through my different life experiences. Throughout the years, transitioning from school to university, I was introduced to key individuals who provided me with various forms of support, which helped me overcome potential barriers and build resilience. Looking back, it isn’t easy to compare the different levels of support from the key members throughout my life as each support figure was present at a different time. Härkönen (2001), who analysed human development according to Bronfenbrenner’s ecological systems theory, emphasises the importance of continuous support of family, educational colleagues, community and the working environment. Härkönen (2001) highlights further that the microsystem is regarded as the most fundamental and crucial support system as it is the most consistent and basic system that safeguards the autistic individual’s emotional, social and physical well-being. This system includes family and friends. Other external support systems outside the home environment, such as the meso-, exo- and potentially macrosystems, include the community of practice, the working environment and international connections. With support from the different systems provided by different individuals and organisations beyond the microsystem, I was able to function.

Adding to the importance of the microsystem, Iivonen et al. (2021) believed that although this internal support network is seen as the most solid and sustainable, it can be disrupted and potentially entangled with tragedy, such as the death of a mother. Relating this to my personal story, the death of my mother was tragic and affected my psychological, emotional, social and physical functioning. However, autism self-advocacy has helped me to develop a robust support system across the micro-, meso-, exo-and macrosystems. The support I received from my wife, my mother-in-law (our carer), my postdoctoral fellow mentor, my psychologist and organisations like the Greenleaf Autism Advocacy Network, the Commonwealth Disabled People’s Forum (CDPF) and the International Council on Development and Learning (ICDL) has been invaluable.

Saarinen (2004) mentioned that Bronfenbrenner’s ecological systems theory is published as a childhood theory but can be related to any individual’s development throughout their life as it is deeply connected to identity development. Relating to my study and the influence of support on my identity development, I see much value in the inclusion of objective self-awareness theory, developed by Duval and Wicklund (1973), which evaluates an individual’s progress, feelings and learning experiences in different contexts. The theory focuses primarily on identity development and how individuals cope and function with expectations in other environments, circumstances and successes (Pinel & Bosson 2013). Taylor and Suthers (2021) believed in the holistic cycle of becoming self-aware of one’s challenges, and through the support that people receive, they can overcome challenges, reduce anxiety and function in a specific environment. The differentiation stage of identity development refers to the moment an individual enters an environment with unfamiliar expectations (Taylor & Suthers 2021). The person simultaneously compares their personality, achievements and character with other individuals in the same environment. This action evokes emotions and feelings of jealousy, disappointment and anger towards others. The second stage of identity development is more general but refers to the transition phase (situation stage) when the individual enters a new stage of their life in which they are required to make informed decisions about life after an extreme setback but are also required to put things in place that will help them to overcome challenges (Silvia & Duval 2001). The recommendations or choices that the individual makes refer to building a system to support and enable them to participate in events that can improve their mental health and self-confidence. This corresponds to the stage of identity versus role confusion (Silvia 2012). After making essential decisions and settling on the recommendations they make, the individual will have gained experience (permanence) and become independent and goal-orientated when it comes to setting personal goals of achievement and making informed decisions (Silvia 2012). The final two goals are to achieve persistence and meta-self-awareness, when the individual will remain consistent and attain personal pleasure and satisfaction (Keats 2010).

In the case of my study, autism self-advocacy changed my life positively. After coming from a very dark place in my life, I needed a solid system that would support me by opening doors to share my story on various platforms locally and internationally. Some of my co-constructors in this study are actively involved in the field of neurodiversity, and through their involvement in my autism self-advocacy journey, they volunteered to participate in my research.

## Research methods and design

I selected critical autoethnography as my methodology to reflect on my self-advocacy journey. This genre is evocative, which enabled me to articulate my experiences and engage in cultural, political and social research (Ellis 2004). By sharing my personal experiences as a researcher, I critiqued cultural beliefs, practices and experiences related to a particular phenomenon, such as my autism self-advocacy journey. The benefit of choosing autoethnography as a methodology, as explained by Butz and Besio (2009), is that the researcher is granted an opportunity to speak from different positions by expressing the richness of the data generated from their fieldwork. By looking at the data ethnographically, the author scrutinises and publicises the data by reworking it to formulate their understanding of the wider world. In other words, the methodology involves deep and mindful self-reflection, balanced by intellectual and methodological rigour, and a desire to seek social justice and improve lives. Importantly, the researcher must be prepared for unexpected emotions to occur when specific memories are revisited. The transformative power of this research process enlightened me and inspired me to continue studying my advocacy journey (Adams, Ellis & Jones 2017). Concerning the study on which this paper is based, the critical incidents were carefully selected to represent pivotal moments that shaped my career as a self-advocate for autism. I revisited these key critical incidents, both positive and negative, to gain a deeper understanding of their impact. Data from these critical incidents were collected through conversations and self-interviews that focused on specific periods during my journey.

Autoethnography as methodology, while inherently personal and subjective, is susceptible to bias (Adams et al. 2017). Although it yields rich insights and diverse perspectives, the findings may lack generalisability because of my unique lived experiences as an autistic individual. The selection of critical incidents was informed by personal reflection; however, incorporating the contributions and memories of co-constructors (whose names are anonymised) introduced additional variability and a unique perspective that validated their inputs. Re-examining sensitive memories related to discrimination and mental health challenges further complicated the objectivity of my research.

In addition, I conceptualised the concept of time mapping to guide my evaluation and planning (reflections). In doing so, I followed six steps. The first step was to identify the criteria for the potential co-constructors with whom to have critical conversations. The second step enabled me to re-look at the data generated from the essential discussions and structure them accordingly. The third step included re-seating and dividing the transcribed data into selected time frames. Lastly, the map was analysed to see how the central ideas were interrelated (Trochim 1989). In a later publication, Trochim and McLinden (2017) acknowledged that ideas or hunches represent these ideas in an objective form in which the constructors’ thoughts are interpreted, and meaning is generated.

**FIGURE 1:**
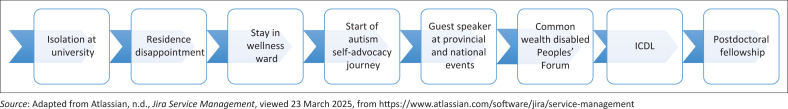
Diagram of the concept map of time with critical incidents.

The content of the concept time map was wholly determined and dependent on the selection of my co-constructors, who were present at different times during my advocacy journey. As the main co-constructor, I needed to be objective about the opinions and views of my co-constructors of that period, as the methodology provided them with a platform to reflect and be honest. I comprehensively reviewed the data generated from the critical conversations and analysed it according to the different time frames I had selected for this study. For preparation purposes, the different discussion points (similar to research questions) were emailed to each co-constructor before the critical conversation to serve as a guide. For each co-constructor, the discussion points were constructed differently because of the different roles that each one had during the specific time frames. The critical conversations were held on platforms such as MS Teams and Zoom at the convenience of the co-constructor. There were four main points of discussion points, each of which led to another based on the perceptions and perspectives of the co-constructors when reflecting on their journey with me. The process described here is one of many ways to accomplish concept mapping of time. Novak (2012) suggested that the advantage of the concept mapping of time is that it is much easier to follow than verbal or written descriptions, and it is more structured; it is referred to as a hierarchical ‘tree’ structure with superordinate and subordinate parts (primary, secondary and tertiary ideas). The map typically begins with a word, concept or phrase representing a focus question and requiring an answer. Link lines are then provided between the hierarchical concepts from top to bottom (Clayton 2006). In Figure 1, the link line (highlighted in blue) links the different sub-themes (influential events) that shaped my advocacy journey.

The two main critical incidents were depicted together with other crucial critical incidents in Figure 1. The link line represents: (1) building resilience during times of disappointment; and (2) the first steps in my journey to self-advocacy for autism, locally and internationally. My introduction to autism self-advocacy with critical moments shaped my identity and built my resilience, which helped me grow into the autism self-advocate I am today.

### Critical incident 1: Building resilience in times of disappointment

Following childhood, I grew up to be an assertive young adult. I always believed that I was not accepted based on my differences, as I was a victim of emotional abuse and was bullied throughout my life. I had a solid support system in the form of my late mother and my father, who supported me academically, emotionally, financially and socially and contributed to building my resilience, especially my mother, who continued to help me throughout my school career to ensure that I coped academically. Even though I was nonspeaking until the age of 15, my mother did not believe in excluding me from the school environment. I did not resist avoiding any form of discrepancy and overcoming the odds against me, as I always thought that I was worth more, that I had a voice and that I needed to exceed my expectations. As Bekhet, Johnson and Zauszniewski (2012) noted, resilience is a dynamic process that encompasses positive adaptation within the concept of adversity, and this corresponded to my motivation to prove critics wrong. Being highly motivated, I was determined to enrol at university, believing it would be the start of my upward journey, although my first 3 years at university were an uphill battle. However, I was determined to persevere and develop my skills, which led to my enrolment in a residence, which was the start of my self-advocacy journey.

#### The residence house: Propelled to self-advocacy, the beginning

As I got used to the residence environment as a third-year student, I challenged my resilience by standing for election to the House Committee. Initially, I was concerned that I was not popular and that I did not have the support of my fellow students because of my differences. Each candidate was required to deliver a short speech during the annual House Committee Circus on the campus of the Faculty of Education. As usual, I discussed my participation with the university psychologist. During our therapy session, he questioned my intention to run and we discussed how I would handle the potential disappointment if not elected. He also warned me of the harshness and judgements that students could face (Gouws 2022).

On the night of the House Committee circus, my anxiety levels were very high, but I knew I had to maintain them. Despite the crowd’s silence, hesitation and laughter, I gave my speech and answered the questions directed at me. When a participant questioned my ability to lead as an autistic person, I responded confidently, highlighting my commitment to making an impact and a difference. I ended my speech with the following lines: ‘Don’t judge a book by its cover’. That response earned me a standing ovation, and I was delighted to finally receive the acceptance I had sought for years. This moment was pivotal and shaped the building blocks of my journey towards self-advocacy for autism. That inner voice was verbalised. Although I was not elected to the House Committee, my satisfaction overruled my disappointment. However, I knew that disappointment in the future would be as hard as the continuous rejection that I experienced, which affected my mental health, so I attempted to commit suicide (Gouws 2022).

#### Attempt to commit suicide

Research by Jackson, Hart and Volkmar (2018) shows that individuals with ASD have higher rates of loneliness, anxiety and depression than typical individuals. In this study, of the 30 individuals with autism from a prominent university in the United States who were interviewed, 17.9% of the total sample thought that it was very likely that they would attempt suicide. Based on my personal experiences as an autistic student living in South Africa, unfortunately, I was part of the attempted suicide statistic, as I reached a moment where I considered ending my life by overdosing on over-the-counter sleeping pills, of which I took six. The hospital psychologist reflected:

‘I can remember that your mom told me that you were studying and that your mental health was not good. You were experiencing depression and anxiety. From what I have heard, you were experiencing a severe rejection from society. You had trouble deciding who you were and where you would fit in. There were some warning signs of suicide that I noticed.’ (Participant 1, Hospital psychologist, Female, Age 40–50 years)

I was rushed to the nearest hospital by my mother and spent 3 days in the ICU because of the development of arrhythmia (a medical condition in which the heart beats at an uneven rate). During my admission to the hospital, the psychologist and my mother decided to admit me to the psychiatric ward because an attempted suicide justified it:

‘At that point, the risk of suicide was on the table, and I decided to hospitalise you because I did not want to take a risk. It was quite a shock to your mother, so she decided it was the best. Luckily, because I worked in the hospital, I could see you every day, and we had pretty intense therapy sessions.’ (Participant 1, Hospital psychologist, Female, Age 40–50 years)

After my short stay in the ICU, I was released, and the hospital psychologist booked me into rehabilitation for 2 weeks (up to 21 days) to receive therapy. During counselling sessions with the psychologist and other activities, we discussed the situation and different coping mechanisms to deal with rejection. To improve my self-confidence, the psychologist recommended that I speak to parents and fellow autistic adults who had experienced the same challenges as me. After numerous sessions, I thought about the psychologist’s suggestion and discussed it with my late mother. We both decided that at that stage, it was the best decision for my mental health and my self-confidence.

### Critical incident 2: The first steps on my journey to self-advocacy for autism, locally and internationally

After returning home from my hospitalisation, I was determined to recover and share my story with other parents. Hours of research led me to discover various organisations that offered training for parents with children on the autism spectrum. I contacted the Greenleaf Autism Advocacy Network, the largest nonprofit organisation representing individuals living with autism in South Africa, seeking guidance and opportunities to contribute to its advocacy events.

One of the first points of contact with an organisation that supports adults and parents with ASD is the Sun Society, nationally and internationally. The therapist named Stefan, who was the lead contact in South Africa, contacted me by email and wanted to meet me and my late mother. During a critical conversation with him, he remembered our initial meeting and made suggestions as to how I could participate in autism self-advocacy and start my healing journey:

‘It was early in 2018. I met you with your mother after she contacted me. Our big question during that time was, ‘How can we support you or find a way to help you?’ It was clear that you were looking for bigger things, and your mom also knew that which encouraged me to introduce you to Christine from the Greenleaf Autism Advocacy Network.’ (Participant 2, Therapist, Male, Age 30–40 years)

During a critical conversation with Christine, the director of the Greenleaf Autism Advocacy Network, she reflected on our introduction meeting, which my late mother attended with me:

‘I wanted to hear from you and appreciated what you had to say. You were kind, open and willing to talk to me. I was hesitant due to my experience with autism self-advocates. As you may know, the others I was involved with were aggressive keyboard warriors. So, for me, it is more about the impact you had on my life, knowing that not all autism self-advocates have the same perceptions and points of view. You were different because you were open to listening.’ (Participant 3, Director, Female, Age 40–50 years)

The Director, Christine, generously extended opportunities for me to participate in autism advocacy, including speaking at small autism awareness campaigns and other events, such as their annual National Autism Symposium. I actively participated in events such as Autism Awareness and Acceptance Month (02 April), contributing through newspaper, television and radio interviews and addressing parents, teachers and autistic adults. Each media appearance and training session significantly improved my self-confidence and sense of worth. My appearance on the fourth season’s second episode of a prominent talk programme in South Africa was a significant milestone. As one of the four interviewees, I shared my personal experiences as an adult on the autism spectrum and discussed my ongoing master’s studies. This exposure amplified my realisation that I had a bigger purpose and a compelling story to share. The overwhelmingly positive response from family members, university staff and the Greenleaf Autism Advocacy Network confirmed the impact of my television interview. It became apparent that overnight I had gained recognition as a prominent autism self-advocate on a national level.

From being exposed to different media and autism self-advocacy platforms, I presented my story at a public event hosted by the province where I reside as part of the autism awareness and acceptance campaign as keynote speaker in 2018 and 2019. Beyond 02 April, these events are significant for the autistic community, as they bring together representatives of various organisations, individuals on the autism spectrum, university officials, school principals and provincial administrators. Sheri, an inclusive education specialist at the Provinces Department, invited me and my mother to present. Sheri recalls the first moments of organising the event and about my aspirations to advocate for autism:

‘I organised an autism awareness event at a school in the province. It was the first event hosted by the province aimed at professionals and parents. On both days, you presented first, followed by your mother. When I first met you on the morning of the event, you were timid and reserved because it was your first time speaking in front of professionals and parents. Hearing your story was vital to me.’ (Participant 4, Inclusive educational therapist, Female, Age 40–50 years)

The event was supported by senior government officials who delivered the annual address to school officials in the district and province. The speech of the Minister of Education was received with the most tremendous respect, as it addressed the crucial issues facing schools and proposed solutions. I had the privilege of meeting the Premier, who commented on my advocacy efforts and boosted my self-confidence. As a first-year specialist teacher, I talked about my experiences as an adult on the autism spectrum. Although the Premier could not attend my session because of other commitments, I received numerous compliments for my presentation, during which I shared personal experiences as an individual on the autism spectrum and its impact on my family. The event and day were memorable for my presentation and the awareness of autism that I created provincially and nationally.

The second conference I attended, which created a platform for me to share my story, was the National Autism Symposium for health care professionals, educators, social workers and fellow autistic adults at the University of the South. This conference had a distinct academic essence. It focused on sharing research findings and lived experiences among autistic adults and parents. Interestingly, this collaborative event was where I met my best friend, another autistic adult, who later became my wife. My presentation, ‘Regulate and Manage Meltdowns’, was a significant part of the symposium. As this was my first major national autism awareness conference, I was very nervous and needed help and guidance to engage with the audience and make an impact. Steve, a fellow presenter at the symposium, provided advice and assistance the night before my presentation in the hotel room where we stayed together. As we reflected on my journey as an autism self-advocate, he said that he believed that my future was bright and that I could make a difference in the field in South Africa:

‘My perspective was based on your qualifications and your life story. By participating in advocacy opportunities, you would get the necessary exposure. Within this environment, you would meet people who work in the same environment and can help you reach that level. Education specialists, therapists, psychiatrists, medical doctors, and all people in the field help expand your network, and you can make a positive contribution by showing people that autism has some positive components. You are an example for many people in the same position as you can follow.’ (Participant 5, Autism self-advocate, Male, Age 50–60 years)

Steve’s opinions could not be closer to the truth as the Greenleaf Autism Advocacy Network asked me to represent South Africa, the organisation and autism at the United Nations in New York the following year at a side meeting hosted by the Commonwealth at the 12th session of the United Nations Congress of State Parties in New York in 2019. This meeting marked the relaunch of the CDPF. It was an experience that I would enter for the first time. To comply with UNCRPD regulations, my brother accompanied me as a personal assistant for the trip. At 26, I was in my second year of autism self-advocacy, starting as a specialist teacher at a mainstream school, completing my master’s degree, and beginning my first year of a doctorate in education. Attending the General Assembly of the Congress of State Parties was overwhelming, especially realising that I was in the same building where Nelson Mandela addressed the United Nations, where he acknowledged the UNCRPD. The schedule was packed, and the participants chose sessions to attend. The CDPF meeting at the Ford Foundation was notable. Representatives from 27 Commonwealth countries attended the meeting, which focused on reviewing constitutions, developing declarations and electing executive committees. I attended executive meetings, recruited organisations to strengthen the CDPF and represented the organisation at key Commonwealth and United Nations meetings such as the Commonwealth Heads of Government Meeting (CHOGM) 2022 in Rwanda. The CDPF nominated me for the Commonwealth Youth Awards based on my contribution to this event.

As an autism self-advocate, my international profile grew tremendously in just a few years with every international speaking opportunity, such as my introduction to the ICDL which came into my life during a critical stage as it was days after my late mother passed away. During this time of grieving, I needed to establish my identity once again and rediscover the importance of autism self-advocacy, and by connecting with ICDL, I would eventually understand the impact that I made in families’ lives. My connection with ICDL eventually started during the coronavirus disease 2019 (COVID-19) pandemic when I applied to participate in their symposium. The chief executive officer of the ICDL, Paul, reached out to appreciate my application and invited me to attend one of their teaching courses. Little did I know that this was the start of a sustainable friendship and the beginning of a life-changing journey. Paul remembers those moments:

‘I remember seeing your conference proposal and immediately sending you an email saying it was accepted. Something about it moved me, and I felt that people needed to hear from you. I remember that at our first Zoom meeting, I thought you were a person who could influence me as a leader. You were valuable and could contribute to the mission of ICDL.’ (Participant 6, Chief executive officer, Male, 50–60 years)

After our first official meeting, Paul and I met virtually at least once every 3 weeks to discuss my experiences as an adult on the autism spectrum in the Global South. The discussion was informal and informative, and Paul and I formed a unique bond. More importantly, it rebuilt my self-confidence, knowing that an international organisation and an influential leader found value in my contributions.

Following my impactful presentation at the first ICDL conference, regular meetings were held with Paul, resulting in an advisory role and an invitation to become a board member and later the selection as President of the Board of Directors. By holding the position of President, I was granted opportunities to feature in international publications such as Forbes Japan. With every opportunity I received from Paul and the ICDL, my confidence grew. Paul witnessed my growth first-hand, and it encouraged him and his fellow board members to nominate and elect me as the President of the Board of Directors. Paul recalls:

‘Watching your voice develop is a fascinating experience. The development of your values and the values that your mother and father instilled in you and all those values remain. When thinking about floor time and *Developmental, Individual Differences, Relationship-Based (DIR)*, we talk about developing the core competencies of human development. It is not about learning skills but more about interacting with stress, working with others, problem-solving, formulating ideas, putting them together, and doing your doctorate work. Watching those competencies develop and be foundationally there because of your experiences and for those to continue to evolve.’ (Participant 6, Chief executive officer, Male, Age 50–60 years)

Advocating for autism has allowed me to speak on international platforms, travel extensively and meet influential politicians and prime ministers. On a more personal note, it has also led to my postdoctoral fellowship and meeting my now-wife. It has also helped me build a support network that has been invaluable in various aspects of my life.

### Ethical considerations

Ethical clearance to conduct this study was obtained from the University of South Africa (UNISA) College of Education Ethics Review Committee (No. 2024/02/14/90548221/43/AM). To protect the identity of my co-constructors, their names and the organisations mentioned have been changed to pseudonyms. The co-constructors were fully informed about the intentions and purpose of the study, and they provided their consent willingly to participate in this study. They also had the freedom to withdraw from the study at any time. As sensitive topics and past experiences were revisited, steps were taken to safeguard everyone’s emotional well-being. Firstly, my co-constructors were notified of the questions ahead of the online conversations for preparation purposes. Secondly, I was mindful of the emotional impact of some of the questions and reaffirmed that professional support was available if needed. Overall, respect and honesty were central to this study as my co-constructors’ contributions were respected and integrated into the research.

## Discussion

Throughout my years as an autism self-advocate, I have come to realise that my advocacy has a much broader purpose than just advocating for myself. Now, I see myself as an advocate for the entire neurodiverse community. In the past, I always aimed for my advocacy to be worth it, even if it inspired just one person, such as a parent at an informational meeting. I have been fortunate to have opportunities for advocacy through organisations like the Greenleaf Autism Advocacy Network, the CDPF and ICDL, both locally and internationally. Through autism self-advocacy, I received opportunities to develop my leadership skills by being elected to the different executive boards. However, it was not until I attended an international autism awareness and acceptance seminar in April 2024 that I fully realised the impact I have had on the autism community and their families. A parent I had never met mentioned during her presentation that witnessing my graduation with a PhD had considerably raised her expectations for her children. This was a significant confidence boost for me. I also realised that my work could be valued in academia as a researcher, especially by publishing articles and first-hand accounts from my perspective as someone from the Global South and a developing country like South Africa. Attending higher-level political meetings at the United Nations and Commonwealth level as a civil society representative has made me aware that the Global North mainly dominates autism research, and the Global South relies heavily on this research. This highlighted the importance of research in gaining the confidence of key role players and politicians to ensure more inclusive policies for individuals living with ASD.

According to Pellicano and Den Houting (2022), autism self-advocates are central figures in the promotion of neurodiversity, challenging the stigma and discrimination associated with autism by reframing the diagnosis as a variant rather than a social problem. The neurodiversity movement is closely linked with social justice and echoes the broader disability and civil rights movements (Hughes 2016). Transitioning to the role of a researcher, I see my contributions to research as eliminating the gap in the Global South by publishing first-hand accounts, but more importantly, how we can make public facilities such as universities more inclusive and accessible for all individuals with autism based on the institutions such as the UNCRPD, the Commonwealth Charter and the global sustainable developmental goals when it comes to reasonable accommodation. Ward and Meyer (1999) explained that self-advocates for autism advocate for their civil rights and educate society to dispel prejudice and discrimination. However, the UNCRPD is a comprehensive international treaty aimed at protecting the rights of persons living with disabilities, especially those with ASD and their carers. There is still a significant gap when it comes to the focus on mental health and the support services available to support autistic individuals, which shows that there is limited data and research available on the mental health and support of autistic individuals in the Global South, especially in South Africa.

## Discrimination

Discrimination is mistreating an individual based on their attributes and actions (Altman 2011). People living with ASD who are different and socially and emotionally vulnerable can become victims of discrimination and victimisation (O’Riordan & Passetti 2006). Research by O’Riordan and Passetti (2006) indicates that individuals with ASD are often discriminated against because of their experiences of inequality. Rosenblatt (2022) emphasised that individuals with autism have a responsibility to advocate against discrimination in various environments. Growing up, I encountered discrimination, including assumptions that individuals on the spectrum could not hold leadership positions. One incident that particularly affected me was when I advised the students in the residence who questioned my candidacy to stand for the House Committee to ‘not judge a book by its cover’, meaning that people are often judged based on their appearance and behaviour. Unfortunately, discrimination against individuals on the autism spectrum is common, leading to mental health challenges and, in severe cases, depression and suicidal thoughts (Chown & Beavan 2012). I can relate to this, as my feelings of not belonging because of societal misconceptions about my differences led me to attempt suicide. Society’s perceptions of people like me align with the medical paradigm, assuming that individuals with disabilities, including those with ASD, can be healed through medical treatment or therapy (Chown & Beavan 2012). Autism self-advocates aim to challenge this medical model and promote a social approach emphasising inclusion, acceptance and reasonable accommodation.

As I delved into the journey, my self-confidence was low. I sought acceptance and validation so I encouraged my late mother to reach out to organisations like the Sun Society and the Greenleaf Autism Advocacy Network to provide me with a platform to share my story and inspire other parents and autistic adults. Each opportunity to speak, locally or internationally, reaffirmed my purpose to inspire and empower others. The feedback from these engagements was overwhelming. It underscored the realisation that beyond academic achievements, I have a meaningful role as an individual on the autism spectrum to contribute to society. Organisations like the Greenleaf Autism Advocacy Network and ICDL have appreciated the personal experiences I brought to each speaking opportunity. After each speaking opportunity, parents expressed gratitude and hope for their children’s future after hearing my presentations, reinforcing the altruistic purpose of my advocacy work.

## The role of autism self-advocacy and the building of resilience

Throughout my life, I have demonstrated resilience by persevering through challenging situations, such as achieving success in school, university and advocacy. This resilience would not have been possible without the support I received from my mother, and now the support I receive personally and professionally from my family and my mentor for my postdoctoral fellowship, which I gained through my advocacy journey. I have transitioned from being assertive and insecure to progressing emotionally and socially, becoming self-aware of the benefits of my autism self-advocacy for my mental health and emotional well-being. My accomplishments, including completing school, attending university, obtaining a PhD, speaking at international conferences and serving on international directorial boards, represent strength, power and promise. The resilience that I have implemented has helped me to develop the ability to cope, overcome and act in times of adversity (Hermann et al. 2024). Hermann et al. (2024) defined resilience as maintaining and regaining mental health despite experiencing hardships and adversity. Through autism self-advocacy, I have gained the skills to organise international autism awareness and acceptance events at a higher political level. I have realised the importance of providing platforms for all individuals living with ASD to have their voices heard, not just higher-functioning adults.

Organisations should recognise the diverse experiences within the autism community and provide equal opportunities for all individuals to advocate for their rights. Following the social model of persons with disabilities, organisations need to create platforms for individuals with different neurodiversities to advocate for their rights. By providing these opportunities, I can now mentor young self-advocates and help them develop their competence, courage and self-advocacy skills. Autism self-advocates can build connections that form a part of their own personal ecosystem, contributing to a sustainable network of care for autistic individuals. It is also crucial for individuals to relate to people who understand autism diagnoses in order to provide the necessary accommodations. This support network aligns with Bronfenbrenner’s ecological systems theory, forming part of the autistic individuals’ micro-, meso-, exo- and macrosystems based on the level of support they need and their self-advocacy efforts. My autism self-advocacy has provided me with the opportunity to build a strong support network and has helped me to formulate my identity. I aim to set an example for others by emphasising the value of support in advocacy efforts.

## Conclusion

This article primarily focuses on my journey from being an autistic adult to becoming an autism self-advocate. This journey has shaped my identity as an autistic adult, postdoctoral fellow and international disability advocate. Through autism self-advocacy, I have found my voice, developed the ability to speak at the highest political level and learnt about disability rights and responsibilities. My experiences have inspired parents to continue supporting their children and raising their expectations for them. At present, my autism self-advocacy supports my research career by providing insights into the topic and helping me gain contacts for potential research initiatives and projects. Furthermore, my autism self-advocacy journey has exposed me to the broader neurodiversity community, shedding light on the daily realities of individuals on the autism spectrum. I have also become aware of the high unemployment and suicide rates among neurodiverse adults in South Africa. Through my participation in higher political meetings, I have understood the importance of sharing the experiences of neurodiverse adults and family members to make policymakers aware of the challenges that those on the autism spectrum face. This article stresses the value of support and shares the experiences of those who have contributed to my advocacy journey.
